# Complex systems biology

**DOI:** 10.1098/rsif.2017.0391

**Published:** 2017-09-20

**Authors:** Avi Ma'ayan

**Affiliations:** BD2K-LINCS Data Coordination and Integration Center; Mount Sinai Center for Bioinformatics; Department of Pharmacological Sciences, Icahn School of Medicine at Mount Sinai, One Gustave L. Levy Place, Box 1603, New York, NY 10029, USA

**Keywords:** complexity, agents, evolution

## Abstract

Complex systems theory is concerned with identifying and characterizing common design elements that are observed across diverse natural, technological and social complex systems. Systems biology, a more holistic approach to study molecules and cells in biology, has advanced rapidly in the past two decades. However, not much appreciation has been granted to the realization that the human cell is an exemplary complex system. Here, I outline general design principles identified in many complex systems, and then describe the human cell as a prototypical complex system. Considering concepts of complex systems theory in systems biology can illuminate our overall understanding of normal cell physiology and the alterations that lead to human disease.

## 1. The science of complex systems theory

Science and technology allow us to understand our environment as well as manipulate it and create new environments and new systems. This led humans to emerge out of nature, and recently to create new complex worlds that highly resemble natural systems [1]. Human-made systems often follow the same design principles governing natural systems. The most important of these design principles is evolution by natural selection [2]. However, human-made systems are not exactly the same as those created by nature. We are gaining an increasing ability to create new complex environments and new machines that perform as well as, or even better than, natural organisms [3]. Man-made complex systems, such as stock markets, or multi-user social online networks, and technologies that can be used to collect and process increasing amounts of data offer us an opportunity to better observe and understand complex systems, natural or man-made. We can increasingly measure the activity of the variables that constitute these systems. This provides a better glimpse at the quantity and connectivity of most variables that control a complex system. When all these variables work together, they make up a system that appears to us as one unit that is alive.

We are beginning to realize that, in general, complex systems, man-made or natural, share many universal design patterns; concepts and principles of design that reappear in diverse, seemingly unrelated systems [4,5]. These design patterns are the essential elements for building successful complex systems that can function, compete, survive, reproduce and evolve for long periods through multiple generations towards increased fitness and overall growth. The science of complex systems theory attempts to gain an understanding about these emerging repeating design principles that reappear in different natural and man-made complex systems and environments [6]. The goal of complex systems science is to define more precisely these properties towards a greater understanding of complex systems as a whole, beyond the understanding of one specific system, or one specific design concept. Better understanding these universal principles will enable us to better digest the rapid changes that occur around us due to technological and social evolution [3]. To study and understand complex systems, when possible, researchers conduct multivariate experiments, recording measurements of the system's variables under a relatively controlled condition to track the system's dynamics under different perturbations over time. These measurements and recordings are used for building models. These models are needed for generating hypotheses consistent with the data. Models attempt to represent the system at a coarse-grained abstraction level, a skeleton of the real complex system under investigation. The process of modelling aims to capture the essence of the complexity, abstracting the real system into a manageable size that is cognitively, mathematically and theoretically explainable. Models that simulate real-world complex systems are built to capture the dynamics and architecture of a system to predict the system's future behaviour and to explain its past behaviour. Such models help us to better understand and potentially fix system failures, such as those happening in disease processes inside human cells. The famous saying about models is that they are all wrong, but some are useful [7], and as such, models play an important role in understanding and taming complex systems. From these models, insightful theoretical rules can be extracted.

However, while we desire to have dynamical models that would explain the behaviour of complex systems, in reality, these models are often too difficult to construct, and when constructed, these models suffer from many shortcomings mainly because of missing information. The problem is both lack of data and data deluge. For dynamical models to be realistic, they need to have accurate initial conditions, exact causality between systems variables [8] and defined kinetics. Such data are often not easily observable. Hence, dynamical models of complex systems suffer from the free-parameter problem where many models can fit the same observed data [9]. The other issue with dynamical models of complex systems is the nonlinearity characteristic of complex systems [10]. Because of the complex relationships between the variables in complex systems, the dynamics of the system quickly become nonlinear and complex, most of which current mathematics cannot explain well. Statistical methods such as correlation analysis, on the other hand, are simpler approaches that today are much more practical [11]. Although correlation-based approaches do not provide full explanation of the system behaviour over time, which is because there are so much data, and because data are missing and inaccurate, finding correlations between system variables provides immediate new knowledge.

In biology, emerging technologies such as deep sequencing of DNA and RNA [12], or mass spectrometry proteomics [13] and metabolomics [14], allow a glimpse into the dynamical state of many components making up the complex systems within human cells. These emerging multivariate biotechnologies, although inaccurate and noisy, help accelerate the discovery of the inner workings of cells in their entirety because they can measure the level of thousands of molecular species all at once, in one experiment. As more knowledge is accumulated about complex systems, such as the human cell, this knowledge can be fed back into the mathematical or computational models to refine them, making them more accurate. This additional information adds more power and value to the models' ability to capture the systems’ functionality in greater detail, and this enables making better predictions about how components and processes of the system come together to enable cellular behaviours such as responses to stimuli that induce cell proliferation, cell growth, cell differentiation/specialization or programmed cell death. The goal is to fill in the missing pieces of the model's puzzle towards better understanding of specific complex systems such as the natural cell. With the accumulation of more data, the scientific method is transforming to rely increasingly on the organization, integration, visualization and utilization of background prior knowledge extracted from large datasets that are composed of measurements recorded from real complex systems variables. This computationally organized background knowledge is used to analyse newly acquired data [15]. As technology advances, recorded data about a complex system's history are accumulating more rapidly than our current ability to store and analyse such data for useful understanding; or in other words, for optimal knowledge extraction. As storage devices are rapidly decreasing in cost, and devices to record almost everything around us are emerging rapidly, we find ourselves surrounded by a sea of data [11]. Such data provide great opportunity to conquer the secrets of complexity but also overwhelm us with bits and bytes of data with no clear meaning. We often find ourselves only using a small fraction of the measured data, only scratching the surface of a mine full of treasures.

## 2. Emerging patterns in complex systems

Different areas of scientific research such as computer science, sociology, mathematics, physics, economics and biology are increasingly realizing the importance of complex systems theory, because the same design patterns and concepts are emerging in these different fields of science. Models that capture complex systems' structure and dynamics are commonly explained by a few governing principles such as survival of the fittest [2], rich-get-richer [16] and duplication–divergence [17]; whereas in fact, there are more forces all acting in concert to shape the structure and behaviour of many different types of complex systems. In combination, these forces can work in parallel, and sometimes counteract one another, to produce the final outcome behaviour of the system that is manifested as continual dynamical and functional structural changes. Different complex systems have slightly different sets of forces, different ingredients that compose their wholes. The proper combination of design concepts and forces, if understood correctly, can lead to an ability to better create, control, predict and fix the complex systems around us, including ourselves and our society, and our natural, economic and technological environments. The human cell, multicellular organisms, economic systems, intricate engineered systems and the Web are all evolving complex systems existing in complex and ever dynamically changing environments. These systems share similar emerging design patterns, the blueprint for generating a complex system. Some of those patterns can be unravelled using modelling.

## 3. Complex environments versus complex agents

**Figure 1.**
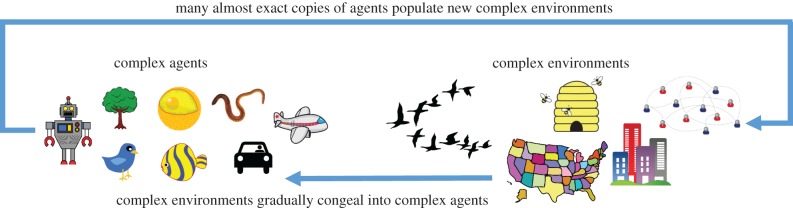
Examples of complex environments: flock of birds, beehive, social networks, cities and states. Examples of complex agents: plane, worm, car, fish, cell, bird, tree, robot. Complex environments gradually tend to evolve into a complex agent. Once many copies of a complex agent exist, these copies can populate a new complex environment. (Online version in colour.)

When using the generalized term *complex systems* and discussing concepts of complex system design, we can distinguish between two main types: complex environments and complex agents. Complex agents are those systems that have clearly defined boundaries, a physical border that encases the system. Complex agents typically have one or a few central processing units, a clock, as well as mechanisms to efficiently obtain and use energy. The agents commonly include sensors and actuators. These types of complex systems interact with their environments through sensors and their actuators, and can typically move, grow, self-repair and self-reproduce. Often, these agents are aware of their existence. Some examples of complex agents are us, our cells, trees, birds, fish, worms, cars, airplanes and some robots (figure 1). The complex agents exist in complex environments, or within other larger encompassing complex agents. On the other hand, complex environments have less defined boundaries. Their governance is also commonly not well defined. These complex systems typically do not have a central processing unit; they do not have a single central brain. Agents in such complex environments are sometimes all similar, or of the same type, or at least have some basic properties in common. Agents in complex environments act as individuals but give rise to the entire dynamics of the system. Examples of complex environments are natural and man-made ecosystems such as flocks of birds, cities, traffic systems, beehives, countries or social networks (figure 1).

The distinction between complex agents and complex environments is blurry because some typical properties of complex environments are present in some complex agents and vice versa. Complex environments are typically populated by complex agents. Intuitively, complex environments grow faster as they become more complex and diverse. On the other hand, complex agents become less flexible as they grow in complexity, so, in principle, evolution slows down as complexity increases for complex agents. As there is a blurry line that separates complex agents from complex environments, it is plausible that these complex systems are just at different stages of their evolution. The complex environments are at the young, newly created stage of a complex system. Over time, these complex environments will begin to congeal, accumulating properties of complex agents one by one as they evolve towards becoming an agent. However, once the system is completely an agent, and there are many almost exact copies of those agents in the environment, these many interacting agents will populate complex environments (figure 1, arrows). This abstract view can be supported by our basic understanding of how biological natural cells came into being, or how multicellular organisms evolved from unicellular organisms. At first, the system was a complex environment where cellular components such as RNA were mixing in the primordial soup [18]. Once more organization had evolved, cells were formed, surrounded by their membranes. Then membranous cells evolved to have sensors and other components that made them become prototypical agents. Once cellular agents existed and proliferated, they started forming multicellular organisms. The first multicellular organisms were created by the same type of cells, but then cell types emerged where different cells assumed different specialized roles. As cells became increasingly specialized, they also became more dependent on one another, ultimately producing a new type of a complex agent, that is, a multicellular organism. Hence, complex environments may be just at an early stage within the complex system evolutionary process, on their way to gradually moving towards becoming a complex agent; once many complex agents of the same type exist in the environment, they can form a new layer of complexity which can serve as a foundation for the next layer.

## 4. Natural versus technological evolution

Complex systems have emerged through natural or man-made evolution. This has produced parallels between natural and technological systems despite their differences. While natural evolution has been evolving for billions of years, man-made technological and economical evolution has made a significant impact on the Earth only in the past few thousands of years [1]. Hence, evolutionary rates are much different when comparing the two types of complex systems: man-made versus natural [3]. Natural evolution needs to wait for random favourable mutations in the DNA of an organism to occur over many generations, whereas in technological evolution new ideas can become new products overnight. It seems that technological evolution is constantly accelerating; it is moving at various rates across the planet, but overall, since the industrial revolution, the rate of complexity of man-made systems seems to be generally accelerating. Different evolutionary rates across the planet are also true for natural evolution. In the rainforest, many species can rapidly emerge because the conditions in that environment are plentiful and favourable for life. There is fresh water, sun and rain, and the temperatures are just right for natural biological life to evolve and thrive. Other areas on the planet such as arid hot or cold deserts do not promote rapid natural evolution, and the emergence of complexity there is slower. Permissive conditions for growth are obvious for natural systems, but less defined for technological evolution. Technological evolution is moving at much faster rates in major cities or on the Web, where interactions between people and the demand for new products are greater than in less habitable regions on the globe. However, there are forces that balance these trends. Geographical diffusion of innovations [19] and the spread of complexity make technological and natural complexity spread to remote places on Earth. Technological complexity is increasingly populating the air, sea and outer space. The sea is full of natural life, but it is not favourable for human life and technological evolution. Space, on the other hand, might be found to be the best place for robots and computers because it is isolated from damaging heat, dust and bacterial agents [20].

## 5. Types of systems versus their instances

A snapshot of a complex system at one particular moment of time captures the systems variables’ state as they are at that time. Such a frozen-in-time state of a system is the manifestation of the instantiation of variables of different types. The distinction between variable types and instantiation of variables, or complex systems types versus actual complex systems, is critical for introducing more clarity. An instance of a variable that is a part of a complex system, or the state of an entire complex system, typically follows the born-live-and-die cycle. On the other hand, the variable, or the complex system *type*, is an abstract representation of the kind of variable or complex system it is. It is not an actual physical entity but a template. Both complex system and variable instances, as well as their types, can evolve. However, actual instances of variables, or entire complex systems, evolve only during the time that they are present, or alive, whereas templates can evolve indefinitely. You are an instance of the complex system that is a human template. The template of a variable, or the type of a complex system, the abstract generalization of the kinds of the real thing, can evolve without a need to be bounded to real existence. The template does not have temporal boundaries. In computer programming languages, the distinction between variables and variable types is clear. Variables can be of different types. Variables are first declared to become instantiated. The variables are then assigned the values that fit their type during program execution. Such values can change while the program is running, and the variables containing the values live within the program for a short period of time when the program runs. Similarly, cells have DNA that serves as a template to produce instances of RNA and protein molecules. Such analogies can help with considering the distinction between an instance and a type, or a template, of a complex system or a variable within a complex system.

## 6. Summary of design principles with initial relations

Complexity theory often focuses on only a few of the design principles of complex systems, most of the time applied to only one real-world complex system: ironically, still reductionism. The reductionist view proposes that complex systems are made of parts, and understanding these parts can lead to the understanding of the entire system [21]. This view dominated science in the past, but it is now accepted that new methods are required to better understand complexity, how the parts come together to give rise to something greater than the parts [22,23]. To achieve such understanding, it may be insightful to examine how design patterns of complex systems are related. To develop intuition about this idea, an initial collection of design principles of complex systems is mentioned below with a brief description of each principle. The next step is to try to identify how these principles are related. The hope is that the relationships between these design principles will become immediately and intuitively obvious. One thing to keep in mind is that definitions of many of these abstract concepts may not be precise; this is a problem because one definition may mean different things to different people. These definitions can surely improve, but making them perfect is challenging, and may require formal mathematical representation. The descriptions of the design principles presented below are abstract but real. So try to not worry for a moment about the specific phrasings of the definitions but the essence of their meaning. Some of these design principles are observed in complex systems in general, covering both natural and technological systems, with some hinted relationships between concepts.

Survival of the fittest is a central design pattern shaping complex systems [2]. This concept is an outcome of competition. Competition is often not fair, where the rich and fit usually become richer or fitter faster than the others [16]. Rich get richer is a growth process where the rich, the ones having many relationships, central, essential and fit, grow faster than the poor, lonely, unfit, weak and less-connected. Complex agents in complex environments commonly also grow by duplication–divergence [17]. Duplication–divergence is a known biological design principle of natural evolution that is also common in technological evolution, economics or on the Web. For example, successful car models, websites and software in general evolve through duplication–divergence. Hence, the successful novel and fit complex agent, organism or product can become an attractor, drawing more connections and copies from it than to it [10]. Sometimes successful novel and fit complex agents emerge from the merger of two existing agents, to form a new innovative and more competitive agent, or product, or organism. Once successful, innovative agents replicate and diversify fast. So innovation plays an important role in the continual evolution of a complex system. Innovations can only become realized on the foundation of already existing, solidified and successful previous innovations [19]. Hence, as mentioned above, complex systems are organized in layers where each layer establishes a solid foundation for the next-order layer to be able to evolve.

Another essential and related principle is information transfer. Information is constantly flowing, commonly compressed, decompressed and translated. Transmitters broadcast information, and then sensors intercept it. Agents in complex systems not only have the ability to passively listen and adapt to their environment, but can also communicate with the environment and change the environment to match their need. Sensors pass information about the state of the environment into the internal central processing centres. Before information is passed to such centres, the signal can be amplified and filtered. In the processing centres, classifiers use the information intelligently, learning from experiences to make optimal decisions about responding and adapting to the state of the environment the next time they are exposed to a previously experienced state. Hence, these classifiers use memory to determine the appropriate future response of the agent. Often this response is simply turning on or off a switch. Sensors, and other components that pass information, implement such switches as well as filters and amplifiers to convert noisy information from the environment to valuable and useful messages, often through the process of discretization or digitization. Tagging, symbolizing, grouping and classifying signals are ways to abstract many similar objects and observations related to forms from the environment into abstract simplified representations. Groups and classes are labelled, converted from their physical reality to symbols encoded into messages. These symbols make it easier for the central processing unit to process information from the environment, and to compute the appropriate response, which involves transmitting information to other complex agents. To compute the right response, internal processing centres use learning, memory and adaptation. The ability to adapt to new environments is critical for the survival of the complex agent living in the complex environment. Robustness to fluctuations and changes in the environment is required for overall fitness and viability [24]. However, a balance between rigidity, robustness and tolerance to change versus flexibility to change is required for providing the necessary level of plasticity for proper adaptation [25]. When learning is successful, responses are commonly automated. Automation is also needed for efficient production. Efficient and sophisticated mechanisms are in place to manufacture many (almost exact) replicas of complex agents and their parts. This allows the cycle of birth–life–death to continue, and for the complex system type to continually proliferate. The birth–life–death concept is related to the observation that complex systems and their parts are dynamically replaced by new parts, while global patterns of the entire complex system and ecosystem remain. For example, proteins in a cell continually turn over, water molecules in a river are not the same but the river stays in constant flow, cars on a highway keep passing, blood cells travel through blood vessels, and people commute back and forth to and from work in and out of a big city; these are only some examples. In some of those cases, these complex agents, or their parts, circulate. This is the case for blood cells, or the people that commute to work, while in other cases the flowing complex agents, or their parts, are completely replaced every time. Hence, complex systems have elaborate and efficient transportation systems that permit the transfer of resources and agents to remote locations quickly and efficiently. Such transportation systems are commonly organized in a tree-like hierarchical structure, where the leaves of the tree, the terminal locations on the tree-like system, often have a unique address encoded in a string of symbols. The hierarchical structure of transportation systems is common in complex systems. To move around, locomotion is necessary. Locomotion is the ability of complex agents to move about in their complex environment. Economic systems rely on planes, ships and trucks to transfer goods and workers from one unique terminal address to another address. Botanic plants lack the ability to move, and this handicap is compensated with an amazing ability to use solar energy, capacity to extract nutrients from the ground, and capability to pollinate and reproduce effectively without the need to travel. Plants and other complex natural systems have seeds that contain compressed information that can be used to create completely new copies of the same complex agents. Such seeds often have mechanisms to travel and diffuse to reach their target for optimal fertilization. They are generated in many copies where each copy is slightly different, and where only a few will be selected to pollinate the next generation.

Notable barriers are present to protect complex agents from other agents and the outside. These containers, or modules, hide internals from exposed externals. The externals have an interface, facilitating the ability to communicate with the environment and other systems, using standard protocols, symbols and flags. Related to this is the plug-and-play design principle that allows reusability and generality. This principle permits complex systems to work together to form higher-order systems. This modularity creates hierarchies. Interacting complex systems have the ability to switch between individual behaviour and behaviour once in a pack. When in a pack, complex systems often form distinct geometrical shapes. Shapes in complex systems commonly tessellate, forming elaborate mosaics [26]. Polymorphic complex systems in a pack behave randomly in parallel but often display amazing synchronicity. Synchronicity can be achieved through governance, for example, by a conductor who signals to an orchestra, but often synchronicity does not require governance in complex systems. Randomness and noise are required for such emergent behaviour. Noise is also required for other aspects of dynamical behaviour that supports complexity and evolution. Noise is a mechanism needed for overcoming being stuck in an evolutionary minimum state. Randomness and noise result in a constant search for homeostasis, but complex systems never settle at a steady state forever [27]. Complex systems continually grow, improve in fitness and increase in complexity because their environment is constantly changing in that direction [28]. Phase transitions happen in short time periods where a system, being in a rather stable state, goes through one small change that induces many changes, turning the system into another new quasi-stable state [10]. Finding an improved fitness state is a design principle directly related to efficiency and energy utilization.

While most processes in complex systems use energy, and where complex agents compete for energy resources, the systems' utilization of energy is more concerned with overall fitness and less with energy conservation and energy efficiency [29]. This is one of many concepts that makes complex systems different from the typical systems that are studied in physics. However, energy conservation and efficiency can help complex systems to better compete. It is interesting that often dead organisms become the energy resource for other organisms, while the most decomposed organic material, crude oil, serves as the major energy source for the initial phase of the technological evolution we see today. Most complex systems typically produce waste; in balanced ecosystems, the waste from one complex system is a resource for another. However, technological man-made complex systems produce waste that is not well recycled. Related to this are feedback loops which are important dynamical structures that set the creation of complex systems in motion. The primordial metabolic soup was made of simple enzymes forming competing feedback loops [18]. Competition involves taking action in markets, where trade makes two or more complex systems winners. Successful trade requires diversity of products and specialization of services. Winners in trade are often the innovators, or the best listeners to innovation. Trade results in cooperation, which can develop into symbiosis: the codependency of two separate complex systems on each other in order to coexist. Unidirectional symbiosis is parasitism. Parasitic complex agents use the success of their hosts for their own survival needs. Successful complex agents must learn how to self-repair and fight parasites, while parasites engage in a game of creative evasion strategies. Parasites sometimes kill their hosts, but not before they replicate and have their copies jump to other hosts, so they can spread.

All the concepts listed above briefly introduce some of the design principles of complex systems with some hinted relationships between them. But more detailed explanations are needed to describe all of these concepts with less ambiguity. In addition, specific examples are required to illustrate how these concepts take shape in real-world natural and technological systems. Such detailed descriptions are beyond the scope of this review; here, however, we are concerned with thinking about how some of those general observations about complex systems apply to human cells and how such a perspective can inform systems biology.

## 7. The human cell: an example of a complex system

The human cell is a complicated living natural machine. Cells that together compose our bodies are a prototypical example of a natural complex system that was evolved and optimized over billions of years. What partially makes human cells a typical complex system is that they are made of many different types of components with many copies of the same components, all working together, interacting in concert and in parallel to form a high-order functional entity that is a part of an organism.

We are made of approximately 50 trillion cells. Almost all these cells contain the same genetic code which is made of long DNA molecules that are strings that hold the template and symbolic instructions that are needed to make an entire organism. Information about how to construct a complete organism is well compressed in the nuclei of human cells. Although the DNA in all our cells is the same, the approximately 400 different cell types constituting our body are markedly different from one another. This is because within each cell type, different sets of genes are expressed. This differential expression of genes is the result of the different extracellular signals that instruct cells how to behave. Cells receive extracellular signals from other cells telling them which genes to express, and in turn, what proteins to make and ultimately how to behave; which cell type they should become. Cells can form elaborate structures and become specialized due to such cell–cell communication protocols that result from either cell–matrix interactions, or from paracrine or endocrine signals coming from other cells carried by small molecules that can pass through the cell membrane, or bind to receptors at the cell surface. These are the complex system sensors. Intracellular cell-signalling pathways are triggered by the complex combination of the extracellular factors all acting in parallel to inform cells about the state of the environment. This form of signalling controls the dynamics of gene regulatory networks that determine the cell's gene expression programme. Cell surface receptors span through the cell's plasma membrane lipid bilayer. This is the barrier of the cell's complex system. These receptors listen to what is happening outside the cell and communicate changes from the environment to components inside cells. When the biochemical concentration of a neurotransmitter in a brain region, or a hormone in the blood, is altered, receptors on the cell's surface can become activated or inhibited. Information of such change is communicated into the cell's central processing unit machinery, which is an intricate signalling network of proteins and metabolites that amplify, filter, process, decode and transmit information. Extracellular small molecules called ligands, such as hormones, neurotransmitters or drugs, bind directly to receptor proteins. The binding of extracellular biomolecules to receptors potentiates receptors to transduce signals by changing the receptors' three-dimensional structure. This change in structural conformation of a receptor results in other proteins present inside the cell, such as enzymes, to change their activity level, for example, by binding or unbinding to the receptors. These intracellular interactions can lead to activation of other enzymes that catalyse biochemical reactions inside the cell. These biomolecular dynamics result in the transfer of information from the outside of the cell into the cell's internal regions. A cascade of biochemical reactions is constantly acting inside cells in parallel where different signalling pathways are constantly becoming activated and deactivated. Hence, information from thousands of receptors of different types, present on the surface of each cell, is integrated to determine the cell's behaviour. This can be achieved by regulating gene expression through activation or inhibition of transcription factors. Transcription factors are proteins that bind to the cell's DNA to regulate gene expression. Other effectors of cell-signalling events are proteins that regulate protein translation, protein degradation, electrical activity modulation through post-translational modifications of channel proteins in the membrane, as well as regulation of several other cellular machineries and organelles inside cells [30].

One of the outcomes of such regulation is the ability of some human cells to crawl [31–33]. The direction and speed of the crawl are determined by the cell signalling network [32], and can be considered one of the cell's actuators. Another organelle that is regulated by the cell signalling network is the mitochondrion. The mitochondria in cells are acting as engines and sensors [34]. They produce the common currency energy sources ATP, GTP and NAD+. These energy-charged molecules can be used by many proteins to perform their work. Interestingly, the mitochondria in cells sense energy levels, and if they receive certain signals, the mitochondria can induce programmed cell death, also called apoptosis [35]. Such altruistic behaviour is initiated by the mitochondria by releasing proteins that trigger signals that lead the cell to commit suicide for the betterment of the entire organism. The mitochondrion's evolutionary origins also exemplify symbiosis. The similarity of the mitochondria to some bacteria that exist today strongly suggests that cells were initially infected with the bacteria, and gradually the bacteria became part of the cell through an evolving endosymbiotic relationship [36].

**Figure 2.**
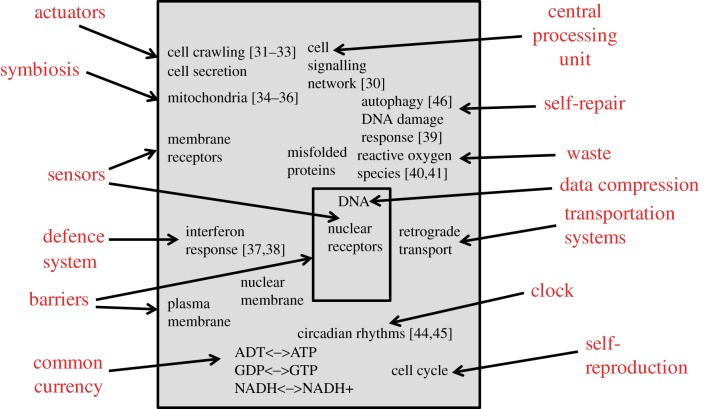
The human cell is a prototypical complex system. In red and outside the box are general complex systems properties. Inside are manifestations of these abstract concepts in human cells. Review articles that further explain some of the subcellular systems mentioned in the figure are as follows: cell crawling [31–33], mitochondria [34–36], interferon response [37,38], cell signalling network [30], DNA damage response [39], reactive oxygen species [40,41], circadian rhythms [44,45] and autophagy [46]. (Online version in colour.)

Programmed cell death is sometimes needed if the cell is damaged or infected. However, before taking such a drastic measure to deal with infection or damage, cells evolved to have defence and self-repair mechanisms. One example of a defence system in human cells is the interferon response to viral infection [37]. Cells have specific receptor and intracellular proteins that can detect viral double-stranded RNA, and signal to the cell signalling network to turn on an immune response. Such an immune response signals to neighbouring cells the news about the infection, as well as triggering an internal reaction to deal with the foreign object in various ways [38]. Similarly, an example of a self-repair mechanism is the DNA damage response, a machinery that can repair double-stranded DNA breaks [39]. The DNA damage response machinery is also linked to the programmed cell death machinery. If the DNA damage is too extensive, the machinery signals to the cell signalling network to activate apoptosis. The DNA damage response machinery is also linked to the cell cycle apparatus, the amazing ability of cells to efficiently self-reproduce a copy of themselves. If DNA damage is detected, the cell cycle programme is signalled to halt. Cell damage can be caused by reactive oxygen species, a by-product of metabolism [40,41]. This can be considered one of the cell's waste products. Cells have developed mechanisms to neutralize reactive oxygen species as well as use them for cell signalling, but at elevated levels these can cause damage and lead to disease. Another example of a cell waste product disposal mechanism is the recent observation that our brain shrinks while we sleep. A recent study suggested that this is needed to remove metabolic toxins accumulated during the day while we were awake and using our brains fully [42]. In Alzheimer's disease, the amyloid plaques that form in the brain could be considered cellular waste that is improperly handled [43]. The circadian cycle in cells is only one of several clocks that are embedded within the cell signalling and gene regulatory networks. These clocks ensure the cyclic regulation of processes that need to be active periodically [44,45]. The above connections between general design patterns observed in many complex systems and those observed in human cells are visually summarized (figure 2). The connections listed are not all inclusive and only made here to illustrate the general concept. It is also expected that as we increase our understanding of the internal components of human cells, many more examples will emerge.

## 8. Conclusion

Cells and their internal constituents are too small for us to observe with the naked eye, and the macromolecular components within cells are only possible to observe with the best microscopes. Until recently, we could only study a few molecular components within a cell in a single experiment. However, with the new biotechnological breakthroughs of the past few decades, we can now understand the inner workings of cells at a greater global scale with refined resolution and detail. This is because these emerging new biotechnologies, for example DNA, RNA and protein sequencing, can measure the level of many molecular species in a single experiment all at once. These technologies produce snapshots of the state of the many variables composing the cellular complex system. This revolution in cell and molecular biology is called systems biology [22], a term that is now interchangeable with big data bioinformatics [47]. It enables the understanding of cell regulation more globally and more holistically. However, to achieve such understanding, new theories explaining how all these parts come together to produce high-order functions are also required. But before such theories can form, we need to be able to handle the masses of data collected using these new technologies. With the rapid reduction in cost for computing and storage, and technologies that permit recording almost everything, we can now track the state of the variables that make up many types of complex systems, over time and under various controlled or natural spontaneous perturbations, including human cells. How many such data do we need to collect in order to build an accurate coarse-grained representation of an entire human cell system? How can we best extract the knowledge nuggets from such data, and make predictions about behaviours and conditions of the system that are not yet measured, or not yet seen? How can we visualize and integrate these high-dimensional data? These are some of the grand challenges facing data scientists today including computational systems biologists.

The field of systems biology is both data-rich and data-poor. It is data-rich because there are mounds of data already collected but needed to be further analysed, and data-poor because the system is so complex and so difficult to observe, and thus currently, the data that we have already collected are clearly insufficient to fully understand the intricate molecular mechanisms that drive human cellular behaviour.

Currently, we do not fully understand all of the molecular details about how cell signalling networks actually integrate and process information to regulate cellular function. Open questions include how the many different ligands, diffusing in the extracellular media, and capable of binding to different and multiple receptor types, initiate intracellular activity changes that result in alternative cellular phenotypes. Until recently, cell and molecular biologists had been using a reductionist approach to study such a complex system. Reductionism in biology has entailed that experimentalists spent their entire scientific careers focusing on analysing only one, or a few, genes and their protein products; where in fact, each mammalian cell has thousands of different types of genes and proteins expressed from these genes, to function altogether simultaneously. All these different types of proteins are working together in concert, influencing each other's activity and level of abundance. However, because such biomolecules are so small, we cannot see exactly how they work, and we have to resort to measuring their activity using indirect methods. Studying only a few genes or proteins by individual laboratories still dominates biomedical research today. The information from the labour-intensive low-throughput single-gene experiments conducted by many different laboratories around the world is continually accumulating. Information from such studies, characterizing individual proteins and their interactions, can be used to reconstruct, through data integration, a more global picture of the cell regulatory puzzle [30]. However, such data collection suffers from research focus biases [48] and reproducibility concerns [49]. However, systems biology approaches are gradually becoming the new standard. The concept of studying systems in biology was introduced before, but then not enough molecular details were available to link molecular interactions to system behaviour [22].

In recent years, much excitement has been generated from the opportunities presented by the promise of artificial intelligence and machine learning, and in particular deep learning. Deep-learning applications to systems biology can indeed accelerate discovery by knowledge imputation [50]. Deep learning can provide answers without the need to know all the details, but can also discover new knowledge that researchers overlooked, similarly to the way a deep neural network discovered new strategies for the game Go, strategies never considered by humans for over 2000 years of mastering the play of this complex game [51]. Technological evolution is also seeing rapid progress due to advances in making deep-learning algorithms more accessible through specialized hardware and open-source easy-to-use software libraries. While these developments enable progress, such progress can sometimes be achieved without fully understanding the implications drawn from a complex systems theory perspective. In this review, I have attempted to further highlight the importance of obtaining a deeper understanding of the human cell as a complex system, as well as other complex systems around us and inside us.

## References

[1] Harari YN 2014 Sapiens: a brief history of humankind. New York, NY: Random House.

[2] Darwin C 1888 The descent of man and selection in relation to sex, vol. 1. London, UK: John Murray.

[3] Kelly K 2010 What technology wants. Harmondsworth, UK: Penguin.

[4] Waldrop MM 1993 Complexity: the emerging science at the edge of order and chaos. New York, NY: Simon and Schuster.

[5] Mitchell M 2009 Complexity: a guided tour. Oxford, UK: Oxford University Press.

[6] Holland JH 1992 Complex adaptive systems. Daedalus 121, 17–30.

[7] Box GE, Hunter WG, Hunter JS 1978 Statistics for experimenters: an introduction to design, data analysis, and model building, vol. 1 New York, NY: Wiley.

[8] Pearl J 2009 Causality. Cambridge, UK: Cambridge University Press.

[9] Prinz AA, Bucher D, Marder E 2004 Similar network activity from disparate circuit parameters. Nat. Neurosci. 7, 1345–1352. (10.1038/nn1352) 15558066

[10] Kaplan D, Glass L 2012 Understanding nonlinear dynamics. Berlin, Germany: Springer Science & Business Media.

[11] Mayer-Schönberger V, Cukier K 2013 Big data: a revolution that will transform how we live, work, and think. Boston, MA: Houghton Mifflin Harcourt.

[12] Sultan M et al. 2008 A global view of gene activity and alternative splicing by deep sequencing of the human transcriptome. Science 321, 956–960. (10.1126/science.1160342) 18599741

[13] Aebersold R, Mann M 2003 Mass spectrometry-based proteomics. Nature 422, 198–207. (10.1038/nature01511) 12634793

[14] Dettmer K, Aronov PA, Hammock BD 2007 Mass spectrometry-based metabolomics. Mass Spectrom. Rev. 26, 51–78. (10.1002/mas.20108) 16921475 PMC1904337

[15] Ma'ayan A, Rouillard AD, Clark NR, Wang Z, Duan Q, Kou Y 2014 Lean big data integration in systems biology and systems pharmacology. Trends Pharmacol. Sci. 35, 450–460. (10.1016/j.tips.2014.07.001) 25109570 PMC4153537

[16] Barabási A-L, Albert R 1999 Emergence of scaling in random networks. Science 286, 509–512. (10.1126/science.286.5439.509) 10521342

[17] Vázquez A, Flammini A, Maritan A, Vespignani A 2003 Modeling of protein interaction networks. Complexus 1, 38–44. (10.1159/000067642)

[18] Oparin AI 1965 The origin of life on the earth. Mineola, NY: Dover Publications.

[19] Rogers EM 2010 Diffusion of innovations. New York, NY: Simon and Schuster.

[20] Teich AH 2008 Technology and the future. Boston, MA: Wadsworth.

[21] Van Regenmortel MH 2004 Reductionism and complexity in molecular biology. EMBO Rep. 5, 1016–1020. (10.1038/sj.embor.7400284) 15520799 PMC1299179

[22] Ideker T, Galitski T, Hood L 2001 A new approach to decoding life: systems biology. Annu. Rev. Genomics Hum. Genet. 2, 343–372. (10.1146/annurev.genom.2.1.343) 11701654

[23] Bar-Yam Y 1997 Dynamics of complex systems, vol. 213 Reading MA: Addison-Wesley.

[24] Barkai N, Leibler S 1997 Robustness in simple biochemical networks. Nature 387, 913 (10.1038/43199) 9202124

[25] Kauffman SA 1993 The origins of order: self-organization and selection in evolution. New York, NY: Oxford University Press.

[26] Ball P 2009 Shapes: nature's patterns: a tapestry in three parts. Oxford, UK: Oxford University Press.

[27] Von Bertalanffy L 1950 An outline of general system theory. Br. J. Philos. Sci. 1, 134 (10.1093/bjps/I.2.134)

[28] Kelly K 2016 The inevitable: understanding the 12 technological forces that will shape our future. Harmondsworth, UK: Penguin.

[29] Ma'ayan A 2012 Colliding dynamical complex network models: biological attractors versus attractors from material physics. Biophys. J. 103, 1816–1817. (10.1016/j.bpj.2012.09.019) 23199907 PMC3491669

[30] Ma'ayan A et al. 2005 Formation of regulatory patterns during signal propagation in a mammalian cellular network. Science 309, 1078–1083. (10.1126/science.1108876) 16099987 PMC3032439

[31] Heidemann SR, Buxbaum RE 1998 Cell crawling: first the motor, now the transmission. J. Cell Biol. 141, 1–4. (10.1083/jcb.141.1.1) 9531543 PMC2132726

[32] Devreotes PN, Bhattacharya S, Edwards M, Iglesias PA, Lampert T, Miao Y In press. Excitable signal transduction networks in directed cell migration. Annu. Rev. Cell Dev. Biol. 33 (10.1146/annurev-cellbio-100616-060739) PMC5792054 28793794

[33] Mayor R, Etienne-Manneville S 2016 The front and rear of collective cell migration. Nat. Rev. Mol. Cell Biol. 17, 97 (10.1038/nrm.2015.14) 26726037

[34] Vyas S, Zaganjor E, Haigis MC 2016 Mitochondria and cancer. Cell 166, 555–566. (10.1016/j.cell.2016.07.002) 27471965 PMC5036969

[35] Green DR, Reed JC 1998 Mitochondria and apoptosis. Science 281, 1309 (10.1126/science.281.5381.1309) 9721092

[36] Gray MW, Burger G, Lang BF 2001 The origin and early evolution of mitochondria. Genome Biol. 2, reviews1018 1011. 10.1186/gb-2001-2-6-reviews1018 PMC138944 11423013

[37] Platanias LC 2005 Mechanisms of type-I- and type-II-interferon-mediated signalling. Nat. Rev. Immunol. 5, 375–386. (10.1038/nri1604) 15864272

[38] Haller O, Kochs G, Weber F 2006 The interferon response circuit: induction and suppression by pathogenic viruses. Virology 344, 119–130. (10.1016/j.virol.2005.09.024) 16364743 PMC7125643

[39] Jackson SP, Bartek J 2009 The DNA-damage response in human biology and disease. Nature 461, 1071–1078. (10.1038/nature08467) 19847258 PMC2906700

[40] Apel K, Hirt H 2004 Reactive oxygen species: metabolism, oxidative stress, and signal transduction. Annu. Rev. Plant Biol. 55, 373–399. (10.1146/annurev.arplant.55.031903.141701) 15377225

[41] Reczek CR, Chandel NS 2017 The two faces of reactive oxygen species in cancer. Annu. Rev. Cancer Biol. 1, 79–98. (10.1146/annurev-cancerbio-041916-065808)

[42] Xie L et al. 2013 Sleep drives metabolite clearance from the adult brain. Science 342, 373–377. (10.1126/science.1241224) 24136970 PMC3880190

[43] Hardy J, Selkoe DJ 2002 The amyloid hypothesis of Alzheimer's disease: progress and problems on the road to therapeutics. Science 297, 353–356. (10.1126/science.1072994) 12130773

[44] Baggs JE, Price TS, DiTacchio L, Panda S, FitzGerald GA, Hogenesch JB 2009 Network features of the mammalian circadian clock. PLoS Biol. 7, e1000052 (10.1371/journal.pbio.1000052) PMC2653556 19278294

[45] Partch CL, Green CB, Takahashi JS 2014 Molecular architecture of the mammalian circadian clock. Trends Cell Biol. 24, 90–99. (10.1016/j.tcb.2013.07.002) 23916625 PMC3946763

[46] Ouyang L, Shi Z, Zhao S, Wang FT, Zhou TT, Liu B, Bao JK 2012 Programmed cell death pathways in cancer: a review of apoptosis, autophagy and programmed necrosis. Cell Prolif. 45, 487–498. (10.1111/j.1365-2184.2012.00845.x) 23030059 PMC6496669

[47] Greene CS, Tan J, Ung M, Moore JH, Cheng C 2014 Big data bioinformatics. J. Cell. Physiol. 229, 1896–1900. (10.1002/jcp.24662) 24799088 PMC5604462

[48] Wang Z, Clark NR, Ma'ayan A 2015 Dynamics of the discovery process of protein-protein interactions from low content studies. BMC Syst. Biol. 9, 26 (10.1186/s12918-015-0173-z) 26048415 PMC4456804

[49] Begley CG, Ellis LM 2012 Drug development: raise standards for preclinical cancer research. Nature 483, 531–533. (10.1038/483531a) 22460880

[50] Xu H, Lemischka IR, Ma'ayan A 2010 SVM classifier to predict genes important for self-renewal and pluripotency of mouse embryonic stem cells. BMC Syst. Biol. 4, 173 (10.1186/1752-0509-4-173) 21176149 PMC3019180

[51] Silver D et al. 2016 Mastering the game of Go with deep neural networks and tree search. Nature 529, 484–489. (10.1038/nature16961) 26819042

